# Diagnostic and Surgical Challenges in Lacrimal Gland Pleomorphic Adenoma: A Case With Prolonged Ptosis and Acute Orbital Changes

**DOI:** 10.1155/carm/4651378

**Published:** 2026-03-06

**Authors:** Khayry Al-Shami, Falah Qudah, Karim Zinhom, Abed Elhakam El-Lababneh, Ziad Haddad, Sondos Qawasmeh, Yahia Ranjous

**Affiliations:** ^1^ Department of Medical Clinical Science, Faculty of Medicine, Yarmouk University, Irbid, 21163, Jordan, yu.edu.jo; ^2^ Faculty of Medicine, October 6 University, Giza, Egypt, o6u.edu.eg; ^3^ Faculty of Medicine, Yarmouk University, Irbid, Jordan, yu.edu.jo; ^4^ Department of Clinical Medical Sciences, Faculty of Medicine, Jordan University of Science and Technology, Irbid, 22110, Jordan, just.edu.jo; ^5^ Department of Surgery, Faculty of Medicine, Damascus University, Damascus, Syria, damascusuniversity.edu.sy

**Keywords:** lacrimal gland tumor, orbital mass, orbitotomy, pleomorphic adenoma, proptosis

## Abstract

**Background:**

Pleomorphic adenoma of the lacrimal gland is a rare benign tumor affecting the orbital area that is usually noninfectious and gradually progressive in nature with painless proptosis.

**Case Presentation:**

We describe a case of a 37‐year‐old woman who presented with an unusual clinical course of ptotic history over five years with proptosis and superolateral orbital fullness emergent in the recent past. MRI showed that the mass originated in the lacrimal gland as a well‐circumscribed and heterogeneous mass without bone erosion or intracranial expansion. Laboratory investigations were normal. A bone window lateral orbitotomy was performed on the patient, where the pseudocapsule was not breached, allowing the excision of the encapsulated tumor in its entirety. Pleomorphic adenoma was diagnosed by histopathological examination. Following surgery, the patient completely recovered with regard to ocular motility, proptosis resolution, and visual function. A seven‐month follow‐up showed the lesion fully resolved, and there was no sign of relapse.

**Conclusion:**

This case demonstrates the significance of the ability to identify abnormal manifestations of lacrimal gland pleomorphic adenoma and underlines its total resection as a method to avoid recurrence and malignant conversion. Surveillance over a long period is essential because it is possible that, after treatment several years ago, recurrence can take place.

## 1. Introduction

Lacrimal gland pleomorphic adenoma (LGPA) is the most common benign epithelial tumor of the lacrimal gland, usually manifesting as a painless, firm, and slowly enlarging mass in the upper outer orbit, often causing proptosis or displacement of the globe. It is most frequently diagnosed in middle‐aged adults, with a higher incidence in females, but can also present in children and, rarely, in the palpebral lobe [1, 2].

The lacrimal gland is a small, specialized gland with limited epithelial tissue compared to the major salivary glands, where pleomorphic adenoma (PA) is more common. That is why LGPA accounts for a small fraction of orbital tumors seen in clinical practice, as it only represents 12%–25% of all lacrimal gland tumors [3]. While the pathogenesis of LGPA is not fully understood, it is thought to arise from abnormal proliferation of both epithelial and myoepithelial cells within the gland [4].

Clinical tests and radiological images (CT and magnetic resonance imaging [MRI]) are used to diagnose LGPA. However, in many cases (22%), misdiagnoses occur, but LGPA can be mistaken for other lesions (such as lymphoma, dacryoadenitis, or carcinoma). In difficult cases, fine‐needle aspiration biopsy (FNAB) can help with diagnosis; nevertheless, incisional biopsy is not recommended because of the possibility of recurrence [5].

The standard treatment is complete surgical excision with the pseudocapsule intact. Preoperative biopsy should be avoided to prevent capsular disruption and recurrence. Recurrence rates are low (< 10%) when excision is complete but increase significantly with capsular violation. Malignant transformation is rare but a serious risk with recurrent or incompletely excised tumors [6].

We describe a case of a 37‐year‐old woman who does not have a history of a previously diagnosed medical illness. She was diagnosed at the clinic with a well‐circumscribed superolateral orbital mass on MRI and a history of recent‐onset proptosis and left upper eyelid ptosis. Histology revealed PA following a lateral orbitotomy for full removal.

The significance of this case lies in its demonstration of an atypical presentation of LGPA that may lead to diagnostic delay. It underscores the key diagnostic and surgical considerations—particularly the role of appropriate imaging and complete en bloc excision in minimizing recurrence and malignant transformation—and contributes valuable evidence to the limited literature guiding early detection, optimal management, and long‐term surveillance of this rare tumor.

## 2. Case Presentation

A 37‐year‐old female patient reported to the ophthalmology clinic, complaining of increased prominence of the left eye (proptosis) in the last 2 months and also had a 5‐year history of ptosis, which promoted further evaluation, with no history of ocular pain, diplopia, or visual loss. The patient had no history of any previous chronic diseases but mentioned two cesarean deliveries as her only surgical history. She was not taking any regular medications and had no history of drug allergies. She is a nonsmoker with regular exercise. A full ocular examination was done, showing that visual acuity was preserved (6/6) in both eyes, with normal color vision (16/16 Ishihara plates), and a mild inferior dystopia was noted in the left eye, with other extraocular movements preserved in both eyes. The pupils were equal and reactive to light, with no relative afferent pupillary defect. The cornea and lens were clear, with deep and quiet anterior chamber bilaterally, and the optic discs and maculae were normal in both eyes. Intraocular pressure was (17–18) mmHg bilaterally, 20 mm in the right eye and 21 mm in the left eye (mild left proptosis), as measured by exophthalmometry. The coexistence of a long history of ptosis with recent onset proptosis, preserved visual function, and normal ocular motility, except for a mild inferior dystopia in the left eye, suggested an orbital space–occupying lesion, most likely in the supertemporal quadrant.

An MRI scan revealed a well‐defined, heterogeneous, enhancing mass that originated from the lacrimal gland in the superolateral quadrant of the left orbit on MRI of the brain and orbits using intravenous contrast.

Dimensions were around 2.4 × 1.9 × 2.2 cm, with no diffusion restriction on DWI/ADC and isointense on T1‐ and T2‐weighted sequences.

**FIGURE 1:**
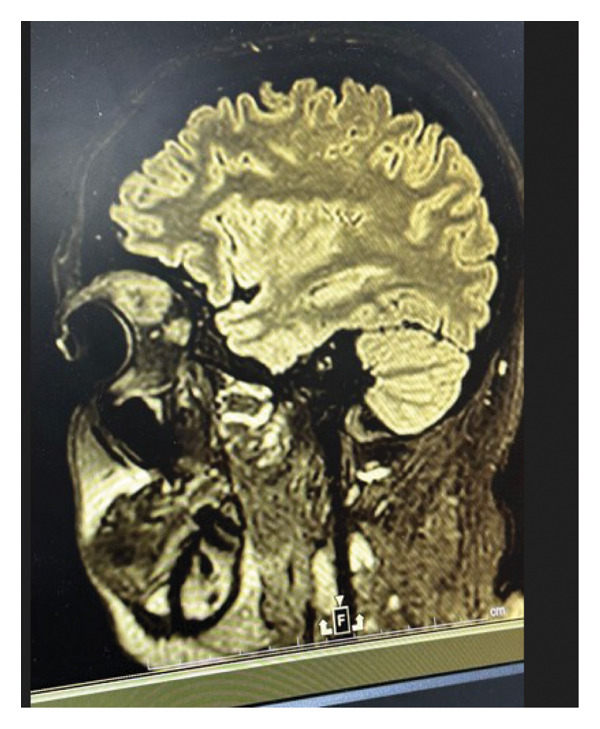
Sagittal T2‐weighted MRI showing a well‐defined orbital mass in the superior extraconal compartment of the left orbit.

**FIGURE 2:**
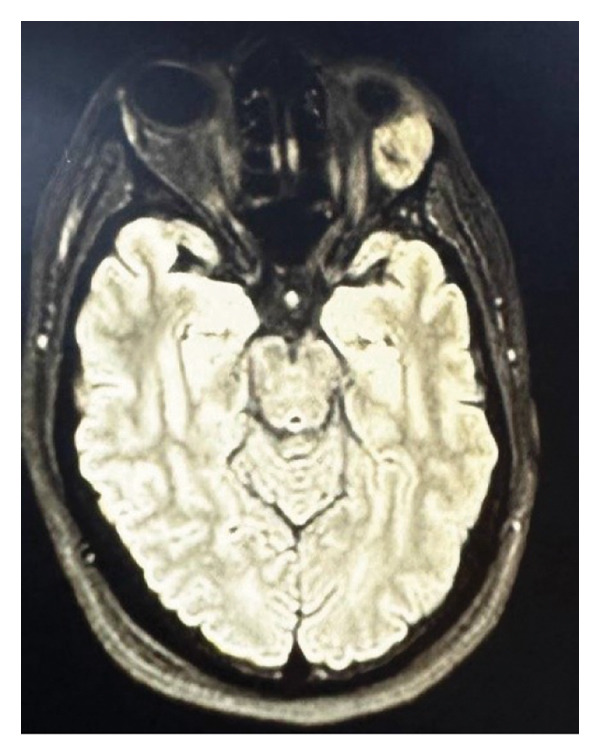
Transverse MRI showing a well‐defined orbital mass in the superior extraconal compartment of the left orbit.

There was no evidence of cerebral extension or bone erosion; instead, the lesion caused mild proptosis by compressing the surrounding extraocular muscles and moving the left optic nerve inferomedially. The globe and orbit on the right looked normal, as shown in Figure 1 and Figure 2.

Preoperative laboratory tests that are routinely performed were within normal ranges. Complete blood count revealed normal hemoglobin levels, white blood cell count, and platelet count. Kidney and liver function tests were within reference limits. All coagulation profile tests were normal, including prothrombin time, partial thromboplastin time, and international normalized ratio (INR), and all the old laboratory tests that were not performed to investigate the mass were normal. The patient’s blood group is A positive.

Laboratory investigations confirmed that there are no systemic abnormalities that might influence surgical or oncologic management of the mass, as represented at baseline with the reference ranges in Table 1.

**TABLE 1:** The laboratory results confirmed a healthy baseline status, appropriate for surgical and oncology management.

Test	Result	Reference range	Interpretation
WBC	8.2 × 10^3^/μL	3.5–11 × 10^3^/μL	Normal
RBC	4.57 × 10^6^/μL	3.8–5.8 × 10^6^/μL	Normal
Hematocrit	38.3%	34%–47%	Normal
MCV	83.9 fL	78–100 fL	Normal
PT	14.2 s	13.4–16.1 s	Normal
PTT	31.9 s	26.5–35 s	Normal
INR	1.05	0.8–1.2	Normal
WBC	7.0 × 10^3^/μL	3.4–9.6 × 10^3^/μL	Normal
Hemoglobin	12.4 g/dL	11.6–15 g/dL	Normal
MCHC	30.9 g/dL	32–36 g/dL	Low
RDW‐SD	48.7 fL	35–46 fL	Slightly high
Creatinine	0.53 mg/dL	0.5–1.2 mg/dL	Normal
Glucose	64 mg/dL	70–110 mg/dL	Low
Electrolytes, LFTs, renal function	Within range	—	Normal

*Note:* The only mild abnormality is a minor deviation (slightly low MCHC and glucose, and a slightly elevated RDW) that was all clinically insignificant.

The excised orbital mass was submitted for histopathological analysis after a complete surgical excision. Microscopic examination proved a diagnosis of PA of the lacrimal gland. Given the clinical and radiological evidence of PA of the lacrimal gland, surgical excision was performed successfully. Following preoperative optimization, under general anesthesia, the patient had a lateral orbitotomy with a bone window in October 2024.

Along the lateral orbital rim, a lateral canthal incision was made and extended to provide sufficient exposure to the superolateral orbit, and a temporary lateral orbital bone flap was created. The tumor was seen to be well‐encapsulated within the lacrimal gland’s orbital lobe. The dissection was done carefully so as not to break the pseudocapsule. The surrounding orbital structures were preserved when the mass was removed in one piece. A solid, hard, lobulated mass that was consistent with a benign lacrimal gland tumor was verified by intraoperative findings. Over the course of the excision, the pseudocapsule stayed intact. The estimated blood loss was less than 100 mL. There were no intraoperative problems.

Layered closure was performedafter repositioning and securing the orbital bone window. It was not necessary to perform a lateral tarsorrhaphy.

At the immediate time postoperatively, the patient developed the expected findings following orbitotomy that were characterized by left upper and lower eyelid ecchymosis and subconjunctival hemorrhage.

Ophthalmic examination was performed at regular intervals (hourly, and then every 12 hours). Visual acuity was preserved (6/6) in both eyes, normal color vision (14–15/5 Ishihara plates), and a mild initial limitation of elevation and supertemporal gaze but subsequently recovered full function. The pupils were equal and reactive to light with no relative afferent pupillary defect. The optic discs and maculae were normal in both eyes.

**FIGURE 3:**
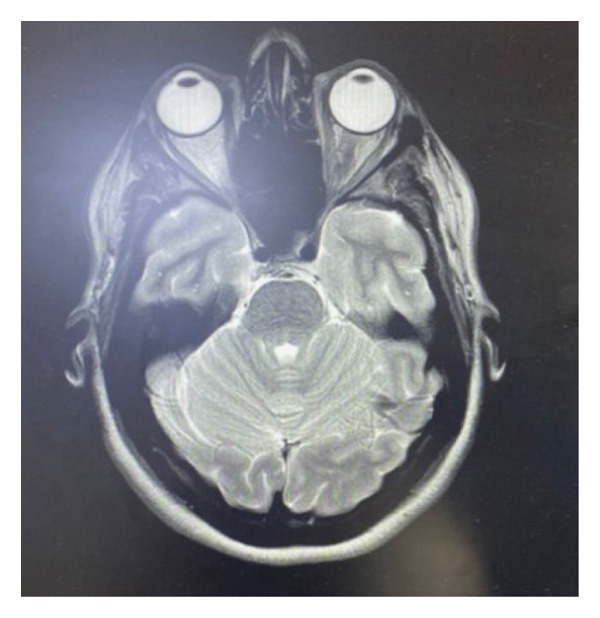
An MRI shows a complete resolution of the previous orbital tumor on brain MRI.

Over the following days, the periocular swelling and subconjunctival hemorrhage gradually resolved. The patient continued to use topical antibiotic ointment (chloramphenicol/phenicol) and lubricating drops (Artelac eye drops), and supportive care was maintained. At her 1‐month oculoplastic follow‐up, she reported resolution of symptoms, with normal ocular alignment and preserved visual function. Schirmer testing demonstrated adequate tear production. At her 7‐month postoperative MRI (May 2025), there was complete resolution of the previously identified orbital mass, with no evidence of recurrence. The extraocular muscles, optic nerve, and globes appeared normal, as shown in Figure 3.

The patient remains under regular follow‐up to monitor for long‐term recurrence or complications.

## 3. Discussion

PA of the lacrimal gland, or benign mixed tumor, is considered a rare orbital tumor with special clinical and surgical considerations. Lacrimal gland tumors represent about 10% of orbital masses overall, and PA is the most common benign epithelial subtype, although it remains rare in incidence [7]. Most ophthalmologists face only a few cases in their careers, underscoring the value of documenting clinical evolution and outcomes in well‐documented patients.

LGPA shows no significant sex predilection and occurs equally in both males and females [8]. Our patient, a 37‐year‐old female, is considered within this expected age spectrum of incidence. The most common presentation is painless, slowly progressive proptosis or superolateral orbital fullness [9]. In our case, the uncommon presentation was a long‐standing 5‐year history of ptosis without proptosis, followed by rapid orbital changes and proptosis over 2 months. This chronic‐to‐acute transformation likely emphasizes tumor enlargement reaching a size to cause orbital compression, resulting in mechanical displacement of the globe and extraocular structures. This evolution represents the slow‐growing but potentially accelerating natural history of PAs and underlines the need for timely recognition before optic nerve compression develops.

MRI is still the gold standard for orbital tumors. PAs characteristically appear as well‐circumscribed, lobulated masses arising from the orbital lobe of the lacrimal gland, with isointense T1 signals, hyperintense T2 signals, and heterogeneous contrast enhancement [10, 11]. These imaging features correlate with their mixed epithelial and stromal histology. Benign diagnosis is supported by the absence of bone erosion, perineural invasion, or irregular margins. The principal differential diagnoses include adenoid cystic carcinoma, lacrimal gland carcinoma ex‐PA, and other epithelial neoplasms, which typically present with pain, bone destruction, or diffuse infiltration [12].

**FIGURE 4:**
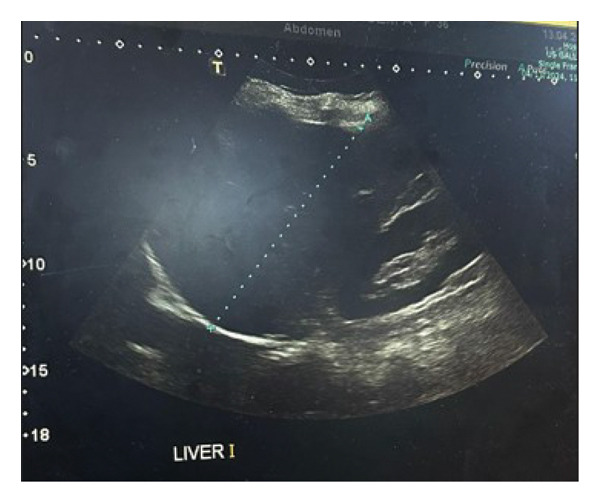
Normal liver ultrasound that excludes any liver metastasis.

An abdominal ultrasound of the liver was performed preoperatively to detect any neoplastic metastasis, as the liver is a common source for tumor metastasis in the body, which appeared to have a normal liver span and smooth hepatic contour, with no signs of any masses, as seen in (Figure 4). Radiological findings, clinical history, and examination are all critical in guiding the management plans.

Histopathology of PA reveals a mixture of epithelial and myoepithelial elements within a variable myxoid, chondroid, or fibrous stroma [13]. Although inherently benign, recurrent disease may harbor atypical features or undergo malignant transformation. Reported rates of malignant transformation vary between 5% and 15% in long‐standing or recurrent cases, with the latency period averaging 10–20 years [14]. Although inherently benign, recurrent PA may display atypical histologic features or progress to carcinoma ex‐PA. Reported malignant transformation rates range from 5% to 15% in long‐standing or recurrent cases, with latency periods of 10–20 years [8]. Therefore, initial surgical management is critical to long‐term outcome. As PA exhibits a varied histopathologic presentation, it may be confused histopathologically with myoepithelioma, adenoid cystic carcinoma, mucoepidermoid carcinoma, and basal cell adenoma [15].

The management of LGPA is maintained by complete excision with an intact pseudocapsule. Biopsy (incisional or fine‐needle) is strongly discouraged, as capsular violation can result in tumor spread, leading to recurrence rates of up to 32% at 5 years [16]. Also, incomplete excision or recurrent tumors markedly increase the risk of malignant transformation into carcinoma ex‐PA [8]. One special article revealed no recurrence in patients with intact excision even after 21 years of follow‐up, compared with late recurrences and malignant transformation in incompletely excised cases [6].

We performed a lateral orbitotomy with a bone window, which is still the gold standard approach for orbital lobe tumors. This surgical technique provides optimal exposure, reduces traction on the globe and optic nerve, and enables en bloc excision while preserving the integrity of the pseudocapsule [17]. Intraoperative handling preserved capsule integrity, ensuring the lowest possible risk of recurrence for the tumor in our patient.

With intact primary excision, long‐term prognosis is excellent, with tumor control rates approaching 97%–100% [6, 7]. However, due to the risk of delayed recurrence or malignant change, extended surveillance is essential, often recommended for at least 10–20 years [10]. Surveillance should include clinical examination and periodic orbital imaging, particularly if new symptoms such as pain, rapid enlargement, or motility disturbance arise.

Our patient’s favorable outcome—with preserved visual acuity, resolution of proptosis, and no recurrence on MRI at 6 months—aligns with the literature on optimal surgical outcomes. Nevertheless, long‐term vigilance is warranted, given that late recurrences have been reported decades after initial excision.

The aim of this case report is to present and analyze a rare clinical case of LGPA and the importance of early recognition to prevent malignant transformation and recurrence. Focusing on its diagnostic challenges and surgical management, this report details the clinical course and multidisciplinary approach in this case. This case has multiple educational benefits, as it is rarely found but important for the differential diagnosis of orbital tumors, being a PA of the lacrimal gland. A distinct clinical evolution as the course of benign orbital tumors is demonstrated by an extended period of ptosis followed by sudden proptosis.

If surgical principles are followed, excellent results can be obtained; however, long‐term follow‐up is mandatory. We aim to contribute to the limited body of literature on this uncommon orbital tumor, to aid clinicians in timely and accurate decision‐making.

## 4. Conclusion

Another critical issue in the differential diagnosis of an orbital mass is LGPA, which is an uncommon complication. The presented case brings to the fore an unusual clinical history, where ptosis was present long before proptosis came in, which should reflect the variability in the manifestations and the importance of an in‐depth assessment of the ongoing eyelid or orbital alterations. MRI is also necessary in diagnosis, although final treatment is based on total surgical excision without a pseudocapsule to avoid the recurrence and malignant transformation. The patient in question had good functional and cosmetic results after undergoing lateral orbitotomy and en bloc resection of the tumor, and no recurrence was observed in further radiologic studies. Surveillance is paramount in the long term, with recurrence and malignant change taking place many years after the beginning of treatment. The case supports the need to identify cases early, do proper surgical planning, and long‐term follow‐up in the treatment of LGPA.

## Funding

No funding was received for this research.

## Ethics Statement

This study is exempt from ethical approval at our institution.

## Consent

Written informed consent was obtained from the patient for publication of this case report and accompanying images. A copy of the written consent is available for review by the editor‐in‐chief of this journal upon request.

## Conflicts of Interest

The authors declare no conflicts of interest.

## Data Availability

The data that support the findings of this study are available from the corresponding author upon reasonable request.
